# Universal screening for cardiovascular disease risk factors in adolescents to identify high-risk families: a population-based cross-sectional study

**DOI:** 10.1186/s12887-016-0548-3

**Published:** 2016-01-21

**Authors:** Michael Khoury, Cedric Manlhiot, Don Gibson, Nita Chahal, Karen Stearne, Stafford Dobbin, Brian W. McCrindle

**Affiliations:** Labatt Family Heart Centre, Department of Pediatrics, The Hospital for Sick Children, University of Toronto, 555 University Avenue, Toronto, ON M5G 1X8 Canada; Heart Niagara Inc., Niagara Falls, ON Canada

**Keywords:** Obesity, Cardiovascular disease, Adolescent, Cardiometabolic risk factors, Cholesterol, Cross-sectional study

## Abstract

**Background:**

Universal screening of children for dyslipidemia and other cardiovascular risk factors has been recommended. Given the clustering of cardiovascular risk factors within families, one benefit of screening adolescents may be to identify “at-risk” families in which adult members might also be at elevated risk and potentially benefit from medical evaluation.

**Methods:**

Cross-sectional study of grade 9 students evaluating adiposity, lipids and blood pressure. Data collected by Heart Niagara Inc. through the Healthy Heart Schools’ Program. Parents completed questionnaires, evaluating family history of dyslipidemia, hypertension, diabetes and early cardiovascular disease events in parents and siblings (first-degree relatives), and grandparents (second-degree relatives). Associations between positive risk factor findings in adolescents and presence of a positive family history were assessed in logistic regression models.

**Results:**

*N* = 4014 adolescents ages 14–15 years were screened; 3467 (86 %) provided family medical history. Amongst adolescents, 4.7 % had dyslipidemia, 9.5 % had obesity, and 3.5 % had elevated blood pressure. Central adiposity (waist-to-height ratio ≥0.5) in the adolescent was associated with increased odds of diabetes in first- (OR:2.0 (1.6–2.6), *p* < 0.001) and second-degree relatives (OR:1.3 (1.1–1.6), *p* = 0.002). Dyslipidemia was associated with increased odds of diabetes (OR:1.6 (1.1–2.3), *p* < 0.001), hypertension (OR:2.2 (1.5–3.2), *p* < 0.001) and dyslipidemia (OR:2.2 (1.5–3.2),*p* < 0.001) in first degree relatives. Elevated blood pressure did not identify increased odds of a positive family history.

**Conclusions:**

Presence of obesity and/or dyslipidemia in adolescents identified through a universal school-based screening program is associated with risk factor clustering within families. Universal pediatric cardiometabolic screening may be an effective entry into reverse cascade screening.

## Background

Landmark autopsy studies have shown that, in children who died accidentally, there was an exponential increase in the extent of their atherosclerotic burden as the number of cardiovascular risk factors increased [1–4]. Many of these risk factors are modifiable, such as obesity, dyslipidemia, hypertension, and abnormal glucose metabolism including increased insulin resistance and diabetes. It has been well established that the presence of these risk factors in childhood increases the incidence of cardiometabolic disease in adulthood [5–7]. In addition, cardiovascular risk factors often cluster within members of families, with both genetic and common environmental/behavioral determinants [8, 9].

Amongst adults, those between 18 and 44 years old have the lowest health-care utilization [10], creating the potential for delayed identification and management of cardiometabolic risk factors and disease. Children, however, typically receive continuous medical care, and recent integrated guidelines have recommended universal lipid screening of pre-pubertal children [11]. Therefore, screening children for dyslipidemia and other cardiovascular risk factors may serve as an entry point to identifying at-risk family members. We sought to evaluate the association between pediatric cardiometabolic risk factors identified through universal school-based cardiometabolic screening and the presence of elevated cardiometabolic risk factors and cardiovascular disease (CVD) in family members.

## Methods

We conducted a population-based cross-sectional study of grade 9 students (14–15 years old) in the Niagara Region of Ontario, Canada during the 2009–2010 school year. The study was undertaken in co-operation with Heart Niagara Inc. Healthy Heart Schools’ Program. This curriculum enrichment program is designed to provide personalized education regarding cardiometabolic risk and healthy lifestyle behaviours, as well as individualized testing, to students in a classroom setting. The program annually targets the entire grade 9 population through their mandatory physical education class (the last school grade where such a mandate exists) in the geographically and administratively defined Niagara Region, Ontario. No students were excluded from participating in the screening. During the 2009–2010 school year, 4104 students participated. All parents of students were provided with questionnaires (described below). Data analysis included all participants who had participated in the screening and had completed the questionnaires. Adolescents presenting with adverse cardiovascular risk profiles were referred back to their primary care provider, where the whole family is encouraged to undergo screening, thus potentially providing a reverse cascade screening tool. The detailed methods of the Healthy Heart Schools’ Program have been previously described [12]. Adolescents provided informed assent and parents/guardians provided written consent to participate in the assessment; the consent included a statement that the participant’s deidentified data may be used for research purposes. Formal ethics approval was obtained by Heart Niagara, Inc. from the research ethics committees of both the Niagara Catholic District School Board and the District School Board of Niagara. The Hospital for Sick Children investigators were approved by Heart Niagara, Inc. for secondary use of deidentified data through a negotiated data-sharing agreement between Heart Niagara, Inc. and The Hospital for Sick Children.

### Data collection

Heart Niagara Inc. staff performed all physical measurements during a scheduled assessment day during usual class time. Height and weight measurements were obtained in a standardized manner. Body mass index (BMI) was calculated (weight in kilograms divided by height in meters squared) and age- and sex-specific percentiles and z scores were determined using the 2006 World Health Organization growth standards [13]. Overweight was defined as a BMI between the 85th and less than the 95th percentile, and obesity was defined as a BMI greater than or equal to the 95th percentile [13]. Waist circumference was measured in a standardized manner, with land marking at the top of the posterior iliac crest with the subject standing. The waist-to-height ratio (WHtR, waist circumference divided by height) was calculated and classified into 3 categories: <0.5, 0.5–< 0.6, and ≥0.6. Previous studies have suggested that a WHtR above 0.5 may be an effective indicator of increased cardiometabolic risk [14–16]. The two remaining categories were based on the methodology of previous studies [17, 18].

Finger stick capillary samples were used to obtain non-fasting levels of total cholesterol (TC) and high-density lipoprotein cholesterol (HDL-C). From this, non-high-density lipoprotein cholesterol was calculated (non-HDL-C, TC minus HDL-C).

Blood pressure was evaluated in a standardized manner as previously described [12]. Systolic and diastolic measurements were converted to age-, sex-, and height-specific percentiles [19]. These values were used to classify the subjects as normotensive (<90th percentile), prehypertensive (90th–< 95th percentile), stage 1 hypertensive (95th–< 99th percentile), or stage 2 hypertensive (≥99th percentile) [19]. If initial measurements were at or above the 95th percentile, the measurements were repeated. If the second measurement was less than the 95th percentile, that value was used and no further blood pressure measurements were performed. However, if the second measurement was at or above the 95th percentile, 6 automated readings were taken at 1-min intervals and the average was calculated.

Students were provided with a questionnaire aimed at reporting whether first degree (siblings and parents) and second-degree relatives (grandparents) had a medical history of dyslipidemia, hypertension, diabetes mellitus, and a history of premature CVD. Premature CVD was defined as any male relative with a heart attack or stroke before 55 years and any female relative before 65 years [12]. The questionnaires were completed at home by the students with help from their parents (for family history questions) and submitted prior to the assessment day.

### Data analysis

Data were analyzed and displayed as means with standard deviations and frequencies, as appropriate. Odds ratios with confidence intervals were used to assess associations between identified cardiometabolic risk factor in adolescents and a positive family history for cardiometabolic risk or early CVD. Only subjects with all completed measurements and a completed questionnaire were included in the analysis. Statistical analyses were performed using SAS statistical software version 9.3 (The SAS Institute, Cary NC).

## Results

Enrollment in the school program for the study period was 4104 adolescents, of which 3467 (85 % of all registered grade 9 students, 50 % male, average age 14.6 ± 0.5 years) adolescents had family history data available. Table 1 shows the descriptive data for the grade 9 participants. Significant differences between male and female students were noted with BMI classification and lipid values. Family history data are shown in Table 2.

**Table 1:** Characteristics of the study population

Variable	Number	Male	Female	p
	3467	1718 (50 %)	1749 (50 %)	
BMI classification (World Health Organization)	3382			0.002
Underweight (<5th percentile)		36 (2 %)	32 (2 %)	
Normal weight (≥5th–<85th percentile)		1042 (62 %)	1157 (68 %)	
Overweight (≥85th–<95th percentile)		251 (15 %)	239 (14 %)	
Obese (≥95th percentile)		349 (21 %)	276 (16 %)	
Waist-to-height ratio	3382			0.24
<0.5		1324 (79 %)	1379 (81 %)	
0.5–< 0.6		281 (17 %)	266 (16 %)	
≥0.6		73 (4 %)	59 (3 %)	
Elevated blood pressure (maximum systolic/diastolic blood pressure)	3359			
Normal (<90th percentile)		1526 (91 %)	1544 (91 %)	0.46
Pre-hypertension (90–< 95th percentile)		76 (5 %)	91 (5 %)	
Stage 1 hypertension (95th–< 99th percentile)		52 (3 %)	44 (3 %)	
Stage 2 hypertension (≥99th percentile)		15 (1 %)	11 (1 %)	
Total cholesterol classification	3061			<0.001
Normal (<4.4 mmol/L)		1282 (84 %)	1122 (73 %)	
Borderline high (4.4–< 5.2 mmol/L)		191 (13 %)	323 (21 %)	
High (≥5.2 mmol/L)		50 (3 %)	93 (6 %)	
Low high-density lipoprotein (HDL-C) (<1.03 mmol/L)	3005	572 (38 %)	279 (18 %)	<0.001
Non-high density lipoprotein (non-HDL-C) classification	3061			0.001
Normal		1224 (80 %)	1149 (75 %)	
Borderline high (≥3.10 to <3.75 mmol/L)		196 (13 %)	268 (17 %)	
High (≥3.75 mmol/L)		103 (7 %)	121 (8 %)	

P values correspond to differences between males and females regarding classification distribution or prevalence of abnormalities

**Table 2:** Family history

	Number	Positive history (%)
Diabetes mellitus-1st degree	3296	291 (9 %)
Diabetes mellitus-2nd degree	3311	1457 (44 %)
Hypertension-1st degree	3229	702 (22 %)
Hypertension-2nd degree	3240	1956 (60 %)
Hyperlipidemia-1st degree	3193	618 (19 %)
Hyperlipidemia-2nd degree	3206	1670 (52 %)
Early atherosclerotic event	3467	1127 (34 %)

**Fig. 1:**
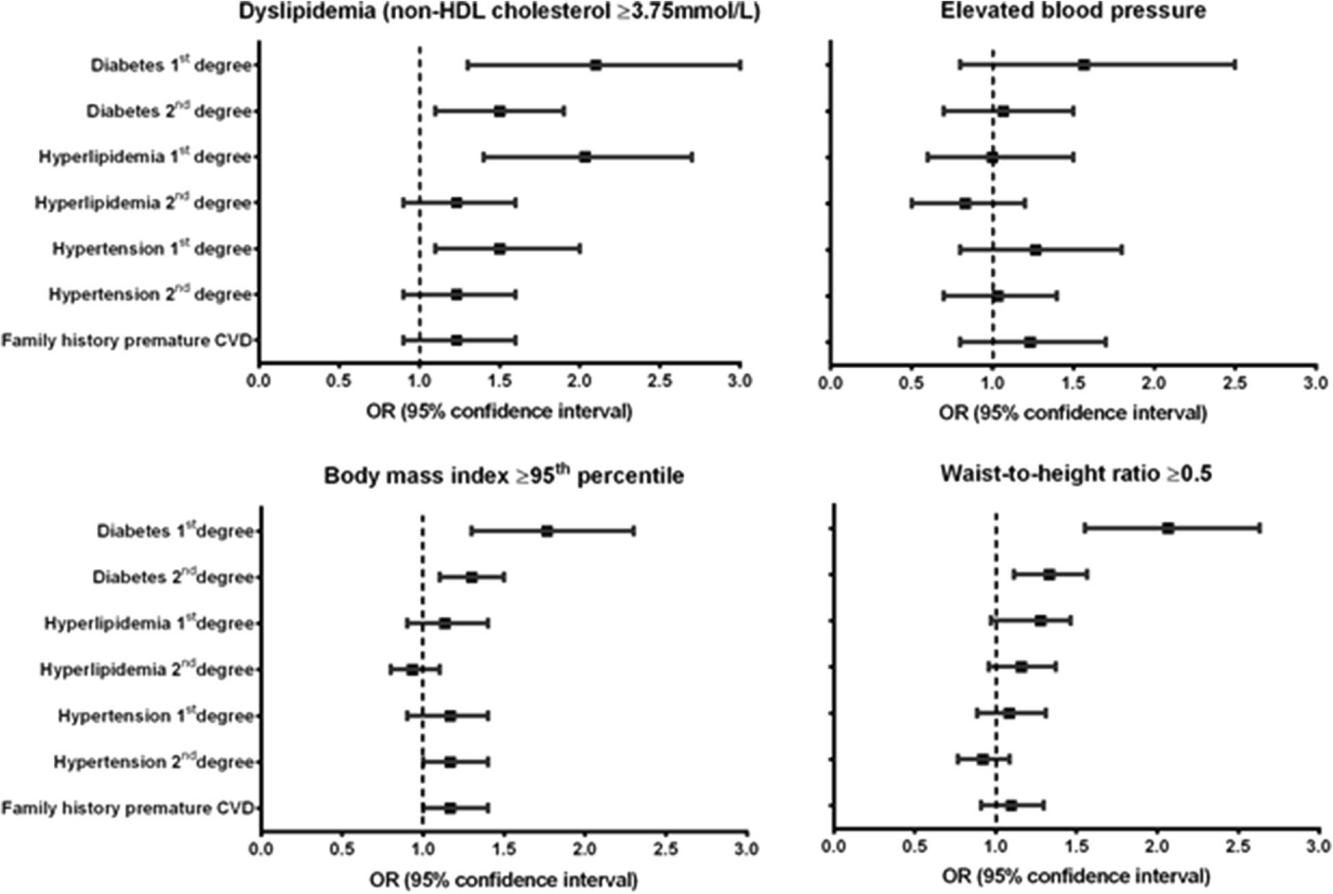
Association between positive screening in the adolescent and odds of abnormalities in 1st and 2nd degree relatives

Forest plots depicting the odds ratios with confidence intervals for a positive family history of diabetes, dyslipidemia, elevated blood pressure, and/or premature CVD in first and second degree family members (based on parental reporting) for a given identified cardiometabolic risk factor in the adolescents are shown in Fig. 1. Adolescents with increased adiposity (BMI ≥95th percentile or WHtR ≥0.5) had increased odds of having a first- or second-degree family member with diabetes. WHtR showed greater odds ratios than BMI. Adolescents with an elevated TC had increased odds of having a first-degree family member with diabetes, dyslipidemia or hypertension. Adolescents with an elevated non-HDL-C had increased odds of having a first-degree family member with diabetes, dyslipidemia, or hypertension, and a second-degree family member with diabetes. Adolescents with elevated blood pressure did not have increased odds of having a family member with a positive family history. None of the measured cardiometabolic risk factors in adolescents were significantly associated with increased odds of having a positive family history of premature CVD.

## Discussion

The findings of our study indicate that cardiometabolic risk factors identified through universal school-based screening of adolescents is associated with the presence of risk factors in family members. The presence of dyslipidemia was associated with a positive family history of diabetes, dyslipidemia, and hypertension while the presence of increased adiposity was associated with a positive family history of diabetes. Adolescents with increased blood pressure measurements did not have increased odds of having family members with increased cardiometabolic risk factors. It should be noted that the elevated blood pressure measurements identified in a universal screening setting are not indicative of a diagnosis of hypertension. This may partially explain why no associations were seen in these subjects. Future studies are required to evaluate this further. No cardiometabolic risk factors in adolescents were associated with increased odds of an early CVD event in a family member. However, obesity, elevated WHtR, and dyslipidemia showed trends towards increased odds.

A number of studies have assessed the association between pediatric cardiometabolic risk factors and the presence of cardiovascular risk factors and disease in family members. Morrison et al.[20] recently evaluated the utility of risk factor screening in 5–19 year-old school children for predicting families at high risk for parental CVD, type 2 diabetes and high blood pressure 26 years later. They found the risk for CVD was greater if children had high TC (relative risk (RR) 1.30) or high low-density lipoprotein cholesterol (LDL-C) (RR 1.26) on screening. Risk for paternal type 2 diabetes was higher in families with pediatric high BMI (RR 1.53). Risk for parental high blood pressure was higher in children who were overweight (BMI ≥85th percentile, RR 1.23), had an elevated LDL-C (RR 1.15), or elevated blood pressure (RR 1.22). In addition, significant child-parent correlations for TC, HDL-C, LDL-C, and glucose were observed. This study concluded that identifying parents, initiated through screening of their children (reverse cascade screening), could possibly identify a cohort of young adults where interventions could be initiated to prevent later CVD, diabetes and hypertension. Our present study showed that parents of adolescents with cardiometabolic risk factors already have increased odds of silent (dyslipidemia and hypertension) and overt (diabetes) cardiometabolic disease. Therefore, not only are family members of children with cardiometabolic risk factors at increased risk of developing cardiometabolic disease in the future, they appear to be at increased risk of having cardiometabolic disease at the time of the school-based screening.

Reis et al.[21] found that parents of children who were obese or had an elevated waist circumference had about 6 times increased odds of having obesity or an increased waist circumference themselves, while children who had hypertension had 15 times increased odds of having a parent with hypertension. These are stronger associations than those observed in our study, possibly due to the small sample size (children and parents from 94 families) and a heterogeneous, high-risk population (52 % of subjects were overweight or obese, 33 % had an elevated waist circumference and 69 % were black). A German study [22] found that children with an elevated waist circumference had 2.55 times increased odds of having a parent with increased waist circumference, while children with elevated blood pressure did not have increased odds of having a parent with hypertension. A child with a raised non-HDL-C had 2.90 times increased odds of having a parent with an increased non-HDL-C. These results are generally similar to the findings in our study, with slightly greater odds ratio values. However, the results of this study may not be generalizable as it used a young (mean age 6.8 years), solely German population with a low incidence of obesity (4.5–4.9 % of children). In addition, similar to Reis et al.[21], this study focused on child-parent correlations for a given cardiometabolic risk factor, whereas our study assessed the odds of a positive family history of dyslipidemia, hypertension, diabetes, and premature CVD for each identified pediatric risk factor.

Muratova et al.[23] performed nonfasting lipid screening of 709 fifth grade children, with confirmation testing for those who screened positive. Of children with confirmed dyslipidemia, 66 % of their parents had confirmed dyslipidemia. The study may not be universally applicable as it took place in a high-risk area, with low education, low socioeconomic status, and low levels of cholesterol screening among adults. In addition, only 36 % of the children who screened positive had confirmatory testing due to logistical issues. Gidding et al.[24] and Polonsky et al.[25] have both shown that children with abnormal lipid levels have an increased incidence of having one or both parents with a lipid disorder. Two studies have shown an increased incidence of premature CVD in grandfathers of dyslipidemic children [26, 27].

Overall, our study shows similar trends to those noted in the literature outlined above, namely that children may be an effective proband for identifying parents and families at risk. However, there are some novel features and findings of the current study. First, our study was performed within an established universal screening program performed in a school-based setting with a large number of subjects. Second, our study assessed a self-reported history of established hypertension, dyslipidemia, and diabetes in family members, showing increased odds of manifest cardiometabolic disease in family members of children with cardiometabolic risk factors. Third, this study showed that children who screened positive for dyslipidemia had increased odds of having parents or siblings with dyslipidemia, hypertension, and diabetes. Identifying a raised risk profile of established cardiometabolic disease in parents and siblings of dyslipidemic children that is this broad is a significant finding. In addition, these results indicate that universal pediatric lipid screening may also identify family members with previously undiagnosed cardiometabolic disease, thus making it a potentially effective entry into reverse cascade screening. Further studies are needed to confirm this.

Recent guidelines [11] have recommended universal lipid screening of all pre-pubertal children between the ages of 9–11, with the aim of early identification of both dyslipidemia of obesity and genetic disorders of dyslipidemia, such as familial hypercholesterolemia (FH). This recommendation has been met with controversy and debate [28–30], with some concerns raised as to whether the available evidence justified the recommendation. The present study has shown a potential added benefit of universal lipid screening that may not have been considered previously: universal school-based lipid screening, when coupled with family history assessment, may identify risk factors clustering within families. Heterozygous FH is relatively common, with a prevalence of at least 1:500 in North America. If left untreated, approximately 25 % of females and 50 % of males will experience a CVD event by the age of 50 [31]. Lipid screening in children can potentially identify previously undetected FH, possibly creating the conditions for an effective and efficient public health initiative. As children typically have parents in an age cohort that typically does not partake in regular health-care visits, identifying children at risk may allow for early identification and intervention for both the child and the parents.

There are a number of limitations that should be considered when interpreting the results of this study. Given the cross-sectional design, only associations, but not causality, can be inferred. As data were collected within a universal screening program, family history data was obtained from questionnaires rather than direct detection of cardiovascular risk factors through measurement of family members. This does not allow for the detection of previously undiagnosed cardiometabolic risk factors or disease in family members. Given that previous studies have shown a large proportion of identified disease in family members to be previously undiagnosed [23, 24], the results of our study may be interpreted as conservative estimates of the true increased risk in family members. However, given that the family history information was obtained from a questionnaire, its accuracy cannot be ensured. In future studies it will be important to perform direct measurements on family members in order to detect previously undiagnosed cardiometabolic risk factors and disease. This will allow a stronger evaluation of the utility of universal pediatric screening as a reverse cascade-screening tool in the detection of cardiometabolic risk factors and disease in family members. Further, the ages of the parents, siblings, and grandparents were unfortunately not available. Ethnicity and pubertal staging data were unavailable. Morrison et al. had previously shown that pubertal status was not a significant explanatory variable for parental outcomes [20]. Finally, the Heart Niagara Inc. screening program currently screens grade 9 students. This is not in keeping with the current expert panel guidelines [11], which suggest the first screening in the pre-pubertal grade 5 population. Future school-based screening studies are needed in this younger population to help further validate the guidelines.

## Conclusions

Adolescents who have cardiometabolic risk factors, identified through universal screening, have increased odds of having family members with diagnosed cardiometabolic risk factors and disease. This indicates that school-based cardiometabolic screening, along with family history assessment, may identify risk factor clustering within families. Future studies are needed to assess the effectiveness of screening pre-pubertal children to help validate recent expert panel guidelines. In addition, further studies are required to establish school-based cardiometabolic screening as an effective reverse cascade-screening tool to detect previously undiagnosed cardiometabolic disease in family members.

## Abbreviations

BMI: body mass index
CVD: cardiovascular disease
HDL-C: high-density lipoprotein cholesterol
LDL-C: low-density lipoprotein cholesterol
non-HDL-C: non-high-density lipoprotein cholesterol
OR: odds ratio
RR: relative risk
TC: total cholesterol
WHtR: waist-to-height ratio
